# Comparative Evaluation of the Effects of Miniscrew and Miniplate Skeletal Anchorage in the Orthopedic Treatment of Growing Class III Malocclusion: A Systematic Review and Meta-Analysis

**DOI:** 10.3390/bioengineering12101065

**Published:** 2025-09-30

**Authors:** Giuliano Irlandese, Giulia Perrotta, Vittoria Marsili, Laura Carboni, Alessio Verdecchia, Enrico Spinas

**Affiliations:** 1 Department of Surgical Sciences, Postgraduate School in Orthodontics, University of Cagliari, 09124 Cagliari, Italy; giuliano.irlandese@gmail.com (G.I.); drgiuliaperrotta@gmail.com (G.P.); vittoriamarsili15@gmail.com (V.M.);; 2 Orthodontics Division, Instituto Asturiano de Odontología, Universidad de Oviedo, 33006 Oviedo, Spain

**Keywords:** maxillary protraction, miniscrew, miniplate, orthodontic treatment

## Abstract

Background/Objectives: Skeletal Class III malocclusion in growing patients presents therapeutic challenges. While traditional tooth-anchored facemask (FM) therapy is widely used, it may induce undesired dental effects. Bone-anchored maxillary protraction (BAMP), using either miniscrews (MSs) or miniplates (MPs), has been proposed to enhance skeletal outcomes and minimize dental compensation. The objective is to compare the efficacy of MS and MP as skeletal anchorage in the orthopedic treatment of the Class III growing patients. Methods: This systematic review and meta-analysis followed PRISMA guidelines. Five databases and manual searches were conducted without restrictions. Inclusion criteria encompassed randomized and non-randomized controlled trials assessing cephalometric outcomes in growing patients treated with MS or MP. Risk of bias was assessed with RoB 2 and ROBINS-I tools, and evidence certainty was evaluated using GRADE. A meta-analysis was performed, collecting all the statistically significant results that emerged in the 11 articles between skeletal anchorage and controls, comparing the values of the MP group with the MS group. Results: Eleven studies (seven MP, four MS) met the inclusion criteria. Both MS and MP groups showed significant maxillary advancement and improved maxillo–mandibular relationships compared to controls. Regarding vertical values, studies have reported contrasting outcomes. Soft tissue improvements were consistent in both MS and MP devices. Statistical analysis has highlighted how MP devices demonstrated more pronounced skeletal effects, while MS systems were associated with more dental effects. Conclusions: MP may be preferable when the aim is to maximize skeletal correction with fewer dental side effects, while MS can be considered in cases favoring less invasive approaches; long-term follow-up and high-quality clinical studies are needed to confirm these clinical assessments.

## 1. Introduction

Skeletal Class III malocclusion is characterized by a sagittal discrepancy between the maxilla and the mandible, resulting from maxillary deficiency, mandibular prognathism, or a combination of both. Most of the time, the malocclusion is determined by maxillary retrusion, followed by a combination of retrognathic maxilla and mandibular prognathism and, less frequently, a mandibular prognathism with orthognathic maxilla [1]. It may present as either a dentoalveolar compensation or a true skeletal imbalance, with varying degrees of functional and aesthetic impact.

The prevalence of malocclusion in the global population ranges from 0% to 26.7%, with considerable variability. This variation is largely attributed to genetic factors, as malocclusion is more common in Asian populations than in Caucasian or African populations [2]. The etiology is complex and multifactorial, involving both environmental and genetic factors, with strong hereditary and familial components.

The treatment of Class III malocclusion presents a significant challenge in orthodontic practice, primarily because mandibular growth is difficult to predict, is largely genetically determined, and is not easily modifiable. Additionally, there is a risk of malocclusion relapsing as patients grow up. Nevertheless, orthopedic treatment involving maxillary advancement during the prepubertal stage may be indicated—either to fully correct the skeletal discrepancy or, in cases where orthognathic surgery will eventually be required, to reduce the extent of skeletal displacement and to improve the stability and predictability of the result. Treatment can be classified based on the timing of intervention: at an early age, by stimulating growth and advancing the maxilla to potentially avoid the need for future orthognathic surgery [3], and after puberty, through orthodontic camouflage or orthognathic surgery, depending on the severity of the malocclusion and the patient’s preferences.

Tooth-anchored face mask (FM) therapy has successfully achieved maxillary advancements in growing patients [4]; however, in addition to the skeletal effects, it presents several dental side effects, including mesialization and extrusion of the molars and protrusion of the upper incisors, increasing in the height of the lower third of the face [5].

In recent years, bone-anchored maxillary protraction (BAMP) has been introduced to maximize skeletal effects while minimizing dental side effects. Two main types of skeletal anchorage are used: modified miniplates (MPs) with intraoral hooks and miniscrews (MSs) that anchored the devices.

Miniscrews are generally inserted into the palatal bone of the maxilla and typically anchored to bone-borne or hybrid expanders on which elastics are connected to the face mask [6] or, less frequently, to a mandibular bar provided with intraoral hooks [7,8].

Miniplates can be placed on the infrazygomatic crest of the maxillary buttress and between the mandibular lateral incisors and canines, where intermaxillary elastics are connected [9], or, alternatively, miniplates can be implanted in the anterior lateral area of the maxilla and connected to a facemask [10,11].

Systematic reviews were identified that compare the outcomes of skeletal anchorage with those of traditional therapies in the management of Class III malocclusion among growing patients [12,13,14]; however, neither of them distinguishes clearly between the results obtained with miniscrews and those with miniplates.

The aim of this study is to compare the efficacy of miniscrew- and miniplate-anchored system devices in the orthopedic treatment of growing patients affected by skeletal Class III malocclusion.

## 2. Materials and Methods

### 2.1. Information Sources and Search Strategy

The present systematic review was performed in accordance with the PRISMA statement of Preferred Reporting Items for Systematic Reviews and Meta-Analyses (PRISMA) guidelines [15]. The protocol was registered in the Prospective Register of Systematic Reviews (PROSPERO) on 5 May 2025 (registration number PROSPERO 2025 CRD420251027914).

The research was performed according to the primary research question: “Are there different effects of using miniscrew or miniplate systems in growing patients with Class III skeletal malocclusion?”

A comprehensive search was carried out on 5 May 2025 on the following databases: PubMed, Embase, Scopus, Web of Science, and Cochrane; no time, language, article type, or additional filters were applied. The detailed search strategy for each database is presented in Table S1 in the Supplementary Materials.

### 2.2. Eligibility Criteria

The PICOS outline (Population, Intervention, Comparison, Outcome, and Study design) of this review is presented in Table S2 in the Supplementary Materials.

Eligible study designs were RCTs and nRCTs, evaluating cephalometric measurements as an outcome after maxillary advancement with miniscrews or miniplates for the experimental group compared to the control group. Eligible participants were required to be children of both sexes in the late mixed or early permanent dentition and prepubertal or pubertal skeletal maturation age with Class III malocclusion.

Exclusion criteria included studies involving subjects with syndromes, congenital anomalies, or systemic conditions that could affect craniofacial development. Furthermore, studies including post-puberal participants were excluded, as well as those lacking skeletal cephalometric measurements or relevant data.

### 2.3. Data Extraction and Synthesis

Two authors (I.G. and P.G.) independently conducted the research process and subsequently screened the obtained results. To evaluate the agreement level among the reviewers, Cohen’s kappa coefficient [16] was calculated. In case of disagreement, a third reviewer (E.S.) was consulted. The authors demonstrated a substantial agreement (Cohen’s kappa: 0.66).

Therefore, an initial screening was conducted based on titles and abstracts, selecting all potentially eligible studies. Subsequently, a second screening was conducted on the full text of the articles according to the established inclusion and exclusion criteria.

For each study included in the research, we collected the following parameters:

(a) Authors, year of publication, and country where the study was conducted.
(b) Study design (type of study).
(c) Sample size of the study group and the control group.
(d) Sample characteristics (age, gender).
(e) Type of orthodontic bone anchorage system (miniscrew or miniplate).
(f) Type of group control.
(g) Treatment duration.
(h) Significant differences between the groups (skeletal, dental, soft tissue parameters).

The primary objective of this study is to compare the effectiveness of skeletal anchorage devices, specifically miniscrews and miniplates, in maxillary protraction therapy for growing patients with Class III malocclusion. The secondary objectives are to assess the skeletal effects produced by the different types of anchorage, to evaluate the dentoalveolar changes associated with treatment, and to analyze the soft tissue modifications that may result.

### 2.4. Quality Assessment

A thorough quality evaluation of the studies was performed using the Cochrane Collaboration’s risk of bias assessment tool (RoB 2) [17] for randomized clinical trials, and the ROBINS-I tool (Risk Of Bias In Non-randomized Studies—of Interventions) [18] for non-randomized clinical trials.

The ROBINS-I tool assesses the risk of bias in non-randomized studies across seven domains: the confounding, selection of participants, classification of interventions, departures from intended interventions, missing data, measurement of outcomes, and selection of reported results.

The RoB 2 scale assesses the methodological quality of randomized clinical trials (RCTs), evaluating five distinct domains: the randomization process, deviations from intended interventions, missing outcome data, measurement of the outcome, and selection of the reported result. Finally, all included studies were evaluated as low risk, some concerns, or high risk. These two scales were utilized to perform the risk of bias assessment.

The GRADE system (Grading of Recommendations, Assessment, Development and Evaluation) [19] was used to rate the quality of scientific evidence and the strength of clinical recommendations in the selected studies.

### 2.5. Meta-Analysis

The main objective of the study is to review and combine the results of a series of works that analyze the effects of orthodontic treatments using miniplates and miniscrews. Through a meta-analysis (MA), the aim is to integrate information from the various studies and thus reach a general conclusion on the research topic.

The variation in each parameter is considered an “outcome” for statistical purposes. The total number of patients included in the studies is 219:147 in the MP group and 72 in the MS group. The results of the estimates, overall effect size, and confidence intervals will be presented in a Forest plot.

The I^2^ index of residual heterogeneity (the percentage of variability in the estimated effect that can be attributed to heterogeneity of the true effects after adjusting the regression) and R^2^ will be calculated, the latter as an indicator of the percentage of total heterogeneity that can be explained by the treatment. Publication bias will be assessed using Funnel plots and Egger’s test.

The level of significance used in the analysis was 5% (α = 0.05). The software used to perform the meta-analysis was R 4.3.1 (R Core Team (2023). R: A language and environment for statistical computing. R Foundation for Statistical Computing, Vienna, Austria. URL http://www.R-project.org/).

## 3. Results

**Figure 1:**
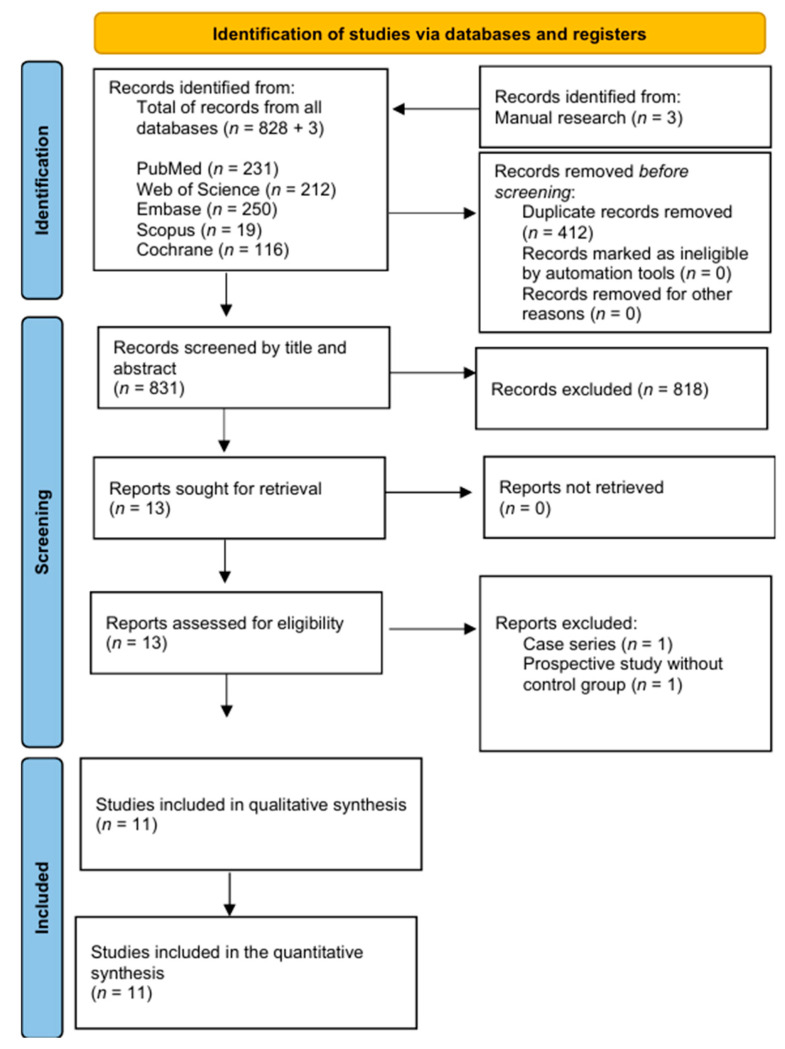
PRISMA flow chart.

The PRISMA flowchart is shown in Figure 1. A total of 828 articles were identified through database searches, and an additional 3 articles were found through manual searching. Prior to screening, 412 duplicate records were removed. After screening titles and abstracts, 818 records were excluded, and 13 full-text articles were assessed for eligibility. Two articles were excluded for not meeting the inclusion criteria (one was a case series, and the other one was a prospective study without a control group). In the end, 11 articles were included in the review.

### 3.1. General Characteristics of Included Studies

The articles were published over a span of 14 years, from 2010 to 2024. The research was conducted across several countries, including the United States and Italy in 2010 [9], followed by studies from Turkey in 2011 and 2017 [11,20]. China contributed publications in 2012 and 2021 [10,21], while Egypt produced several studies in 2016, 2018, and 2023 [7,22,23]. In 2017, a collaborative work came from both Egypt and the United States [24]. Japan contributed in 2020 [25], and the most recent publication, from 2024, came from the United Kingdom [26].

As shown in Table 1 the included studies comprised six RCTs [7,10,21,24,25,26] and five nRCTs [9,11,20,22,23]. The experimental groups encompassed 219 patients aged 9.0 to 13.6 years, while the control groups included 208 subjects aged 8.8 to 13.1 years. Gender distribution was balanced across groups, with 119 males and 100 females in the experimental cohorts, and 118 males and 90 females in the controls. Treatment duration for both miniplate and miniscrew protocols ranged from approximately 6 to 18 months.

### 3.2. Qualitative Analysis

A qualitative analysis of the studies was performed by comparing separately the results obtained between MP anchorage and tooth-borne devices and untreated controls, and between the MS system and tooth-borne devices and untreated controls, reporting all the values that were found to be statistically significant (Table 2 and Table 3).

#### 3.2.1. Comparison: Miniplate vs. Tooth-Borne

Sagittal Skeletal Parameters

Sar et al. (2011) [11] reported significant maxillary advancement (SNA, A-VR, NPerp-A, and Cd-A), mandibular posterior displacement (SNB and Pg-VR), and improved maxillo–mandibular relationship (Wits) in the miniplate group compared to controls.

Liang et al. (2021) [10] also found greater sagittal improvement in the miniplate group by maxillary advancement (SNA, ANS-V plane, and A-V plane) and maxillary length (PNS-ANS).

Vertical Skeletal Parameters

In the study by Sar et al. (2011) [11], the tooth-borne group showed greater posterior mandibular rotation (SN^GoGn and HR^GoMe) and increased anterior and total facial heights (ANS-Me and N-Me).

Liang et al. (2021) [10] reported clockwise palatal plane rotation (PP-SN) in the miniplate group.

Dental Parameters

Sar et al. (2011) [11] observed greater proclination and protrusion of the upper incisors (U1i-VRmx, U1^HRmx, and U1^PP) and mesialization of the upper molars (U6-VRmx) in the tooth-borne group, while the miniplate group showed more retrusion and retroclination of the lower incisors (L1i-VRmd).

Liang et al. (2021) [10] found similar findings, with reduced upper molar extrusion (U6-FH and U6-PP), retraction, and retroclination of lower incisors in the miniplate group (L1-GoGn and MidL1-V plane).

Soft Tissue Parameters

Sar et al. (2011) [11] reported increased upper lip protrusion in the miniplate group (A’-VR and UL-VR).

#### 3.2.2. Comparison: Miniplate vs. Untreated

Sagittal Skeletal Parameters

Sagittal changes were reported in five studies.

Sar et al. (2011) [11] reported significant maxillary advancement (SNA, A-VR, NPerp-A, and Cd-A), mandibular posterior relocation (SNB, B-VR, NPerp-Pg, and Pg-VR), and improved maxillo–mandibular relationship (ANB, (A-VR)-(B-VR), and Wits) in the miniplate group. They also found an increase in mandibular length (Cd-Gn) in the control group.

De Clerck et al. (2010) [9] reported significant maxillary advancement, as evidenced by increased A-Vert, Ptm-VertT, Or-VertT values, and maxillary length (Co-A) in the sample group. Improvements in maxillo–mandibular relationship were also observed, with increases in Wits appraisal and maxillo–mandibular differential (Mx-Md diff), accompanied by posterior repositioning of the mandible (decreased B-VertT and Pg-VertT) and a minor mandibular length (Co-Gn).

Similarly, Eid et al. (2016) [22] found significant increases in maxillary advancement (SNA and N-A-Pog) and improvements in maxillo–mandibular relationship (ANB), and greater mandibular retrusion (SNB). Bozkaya et al. (2017) [10] reported significant increases in maxillary advancement (SNA, Co-A, A-y, and ANS-PNS) and improvements in maxillo–mandibular relationship (ANB, Wits, and ANS-PNS/GoGn) in the miniplate group. They also found a greater mandibular posterior relocation (SNB and B-y).

Elnagar et al. (2017) [24] showed similar results, including increased maxillary advancement (SNA, A-VertT, Or-VertT, Ptm-VertT, SNO°, and A-NPerp), maxillary length (Co-A), and improved maxillo–mandibular relationship (Wits and ANB) in the miniplate group. Mandibular posterior relocation (B-VertT, Pg-VertT, and SNB) was also more evident in the miniplate group.

Lastly, Mandall et al. (2024) [26] reported a significant improvement in the ANB angle.

Vertical Skeletal Parameters

Vertical changes in the MP group were reported in four studies.

In Sar et al.’s (2011) [11] study, the MP group showed greater posterior mandibular rotation (SN^GoGn, BaNa^PtGn, mandibular rotation, and HR^GoMe), greater anterior rotation of the maxilla (HR-PNS and HR^PP), and an increase of anterior and total facial heights (ANS-Me, N-Me, and S-Go/N-Me).

De Clerck et al. (2010) [9] observed a reduction in mandibular divergence (ML-SBL and Co-Go-Me) and a clockwise rotation of the palatal plane (NL-ML).

Bozkaya et al. (2017) [20] found a significant increase in total anterior facial height (ANS-Me), clockwise mandibular rotation (SN^GoGn, B-x, and Co-x), and rotation of the palatal plane (A-x).

Elnagar et al. (2017) [24] reported similar results: more clockwise rotation of the mandible (SN^GoGn) but a significant reduction in the gonial angle (Ar-Go-Me).

Dental Parameters

Dental side effects were generally less pronounced than in tooth-borne protocols.

Sar et al. (2011) [11] observed greater proclination and protrusion of the upper incisors (U1i-VRmx, U1^HRmx, and U1^PP) in the untreated group and a greater retroclination and retrusion of lower incisors (L1i-VRmd, L1^HRmd, and L1^MP), while the miniplate group showed an increase in overjet.

De Clerck et al. (2010) [9] documented proclination of the lower incisors (increased L1^MP), along with increases in overjet and overbite.

Eid et al. (2016) [22] found significant upper incisor proclination and protrusion (U1^SN, U1^PP, U1^L1, U1-NPog).

Bozkaya et al. (2017) [20] reported more retrusion and retroclination of the lower incisors (L1-NB, L1^NB, and L1-y2), increased overjet, and a reduction in overbite.

Elnagar et al. (2017) [24] also found increased overjet and decreased overbite.

Mandall et al. (2024) [26] found only an increase in overjet.

Soft Tissue Parameters

Soft tissue improvements were observed in five studies.

Sar et al. (2011) [11] reported increased upper lip protrusion (A’-VR and UL-VR), a more significant chin retrusion (B’-VR and Pg’-VR), and greater lower lip retrusion (LL-VR) in the miniplate group. On the other hand, the sample group presented an increase in lower (Sn-Me’) and total (N’Me’) soft tissue facial height.

De Clerck et al. (2010) [9] reported increased protrusion of the upper lip (A’-VertT, Sn-VertT, Ulip-VertT) and retrusion of the lower lip (Llilp-VertT) and soft tissue chin (B’-VertT, Pg’-VertT).

Bozkaya et al. (2017) [20] confirmed these trends, reporting increased upper lip protrusion (UL-S line) and retrusion of the lower lip (LL-S line).

Elnagar et al. (2017) [24] similarly observed upper lip advancement (A’-VertT), lower lip retrusion (B’-VertT), and soft tissue pogonion retrusion (Pg’-VertT).

Soft tissue parameters were not reported by Eid et al. (2016) [22] or Mandall et al. (2024) [26].

#### 3.2.3. Comparison: Miniscrew vs. Tooth-Borne

Sagittal Skeletal Parameters

Seiryu et al. (2020) [25] reported significantly greater forward displacement of the maxilla in the miniscrew group, reflected in increased SNA and SN-ANS values. The ANB angle also increased more markedly, indicating improved maxillo–mandibular sagittal relationships.

In contrast, Ge et al. (2012) [21] did not observe statistically significant differences in sagittal skeletal outcomes between the groups.

Vertical Skeletal Parameters

Ge et al. (2012) [21] and Seiryu et al. (2020) [25] did not report significant vertical skeletal differences between the groups.

Dental Parameters

Ge et al. (2012) [21] observed greater upper incisor proclination (U1^SN), protrusion (U1-VR), and upper molar mesialization (U6-VR) in the tooth-borne group.

Seiryu et al. (2020) [25] also reported increased upper incisor proclination (U1^SN) in the tooth-borne group.

Soft Tissue Parameters

Ge et al. (2012) [21] did not report significant values between the groups, while Seiryu et al. (2020) [25] reported no parameters.

#### 3.2.4. Comparison: Miniscrew vs. Untreated

Sagittal Skeletal Parameters

Eissa et al. (2018) [23] reported a significant forward displacement of the maxilla, with increased SNA angle and enhanced maxillo–mandibular relationships (ANB and Wits) in the miniscrew group compared to controls.

Kamel et al. (2023) [7] confirmed these findings, documenting an increased maxillary advancement (A-VR, A-NPerp, and SNA), maxillary length (Co-A), and improvements of maxillary–mandibular relationship (ANB and Wits) in the sample group. Moreover, a greater mandibular posterior relocation (SNB, B-VR, and Pg-VR) in the miniscrew group was reported.

Vertical Skeletal Parameters

Eissa et al. (2018) [23] reported a more counterclockwise rotation of the occlusal plane (SN^Occ) in the sample group.

Kamel et al. (2023) [7] found a significant decrease in the gonial angle (Ar-Go-Me) but an increase in lower anterior facial height (ANS-Me) in the miniscrew group.

Dental Parameters

Eissa et al. (2018) [23] found a significantly greater upper incisor proclination and protrusion (U1^NA and U1-NA), upper incisor intrusion (HRL-U1), and lower incisor retroclination and retrusion (L1^NB and L1-NB) in the sample group. Additionally, upper molars were mesialized (VRL-U6), while lower molars showed distalization and intrusion (VRL-L6 and HRL-L6). A significant increase in overjet was also found in the sample group.

Kamel et al. (2023) [7] confirmed upper incisor proclination (Is-PP), protrusion (Is-yMx), upper molar mesialization and extrusion (Ms-yMx and Ms-xMx), and increased lower incisor protrusion and proclination (IiyMd and IiMP) in the miniscrew group. Additionally, overjet increased, while overbite decreased significantly.

Soft Tissue Parameters

Eissa et al. (2018) [23] reported increased upper lip protrusion (UL-E plane), lower lip retrusion (LL-E plane), and a significant reduction in the nasolabial angle (NLA) in studies using miniscrews.

Kamel et al. (2023) [7] corroborated these results, with increases in upper lip protrusion (A’-VR and Ls-VR), lower lip retrusion (Li-VR), and retrusion of the soft tissue pogonion and point B (Pg’-VR and B’-VR) in the sample group.

#### 3.2.5. Risk of Bias in Individual Studies

**Figure 2:**
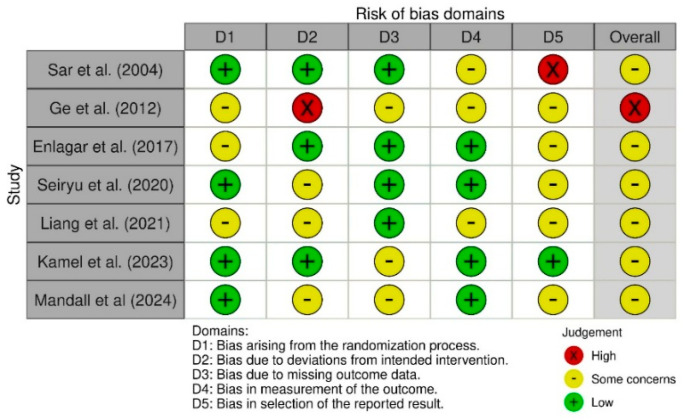
Risk of bias assessment for RCT performed with ROB 2 tool [7,10,11,21,24,25,26].

**Figure 3:**
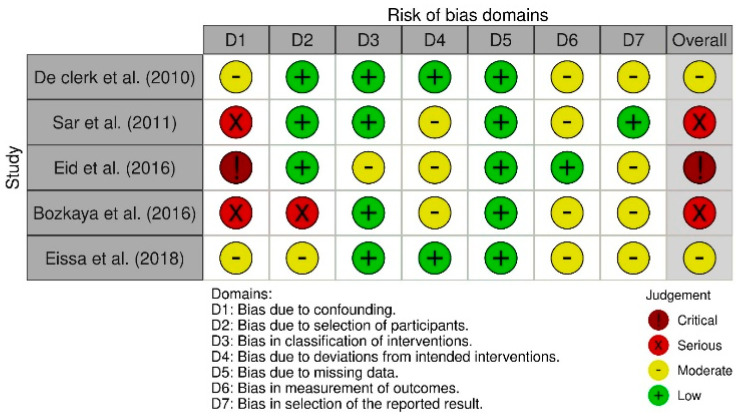
Risk of bias assessment for nRCT performed with ROBINS-I tool [9,11,20,22,23].

The selected studies were evaluated with the ROBINS-I tool for non-randomized clinical trials (n-RCTs) and the ROB 2 tool for randomized clinical trials (RCTs). Figure 2 displays the results generated with ROB 2, whereas Figure 3 presents those obtained with ROBINS-I.

The included studies were appraised using the ROBINS-I tool for non-randomized clinical trials (nRCTs) and the RoB 2 tool for randomized clinical trials (RCTs). Figure 2 presents the RoB 2 results, while Figure 3 displays the ROBINS-I findings.

For the RCTs, the RoB 2 assessment indicated “some concerns” for most articles and a “high” risk of bias for one article. Domain-level analysis showed low to moderate risk in Domains D1, D3, and D4, and a higher risk (ranging from low to high) in Domain D2 (bias due to deviations from the intended intervention) and Domain D5 (bias in the selection of the reported results).

For the nRCTs, the ROBINS-I evaluation identified an overall “critical” or “serious” risk of bias in three studies, and “some concerns” in two studies. The three studies rated as having a critical or serious overall risk also received the same judgment for Domain D1 (bias due to confounding). Domain D5 (bias due to missing data) showed a low risk across all studies. The highest risk was observed in Domain D1, ranging from moderate to critical. In Domain D2 (bias due to selection of participants), the risk was low in three studies, moderate in one, and serious in one. Domains D3 (bias in classification of interventions), D4 (bias due to deviations from intended interventions), D6 (bias in measurement of outcomes), and D7 (bias in selection of the reported result) showed risks ranging from low to moderate.

According to the GRADE approach, the overall certainty of evidence for miniscrew studies was rated as “high” in three of the four trials [7,21,25] and one study by Eissa et al., 2018 [23] rated it as “moderate.” Among studies on miniplates, three of the seven investigations by Liang et al., 2021 [10], Elnagar et al., 2017 [24], and Mandall et al., 2024 [26] achieved a “high” certainty rating, while the remaining four, Sar et al., 2011 [11], De Clerck et al., 2010 [9], Eid et al., 2016 [22], and Bozkaya et al., 2017 [20], were rated as “moderate.”

Detailed GRADE tables for both miniplate and miniscrew studies are available in the Supplementary Materials (Tables S3 and S4).

### 3.3. Quantitative Results: Meta-Analysis Outcomes

The researchers conducted an exhaustive review of the literature, resulting in a final selection of 11 studies, 7 with MP and 4 with MS. Two of these include two subgroups each and can therefore be considered as independent studies (a total of 13 comparisons). Each of these studies aimed to compare a treatment group (MP or MS) with a control group (either untreated or with dental anchorage). The cephalometric parameters reported in the articles can be grouped into four categories: sagittal skeletal, vertical skeletal, dental, and soft tissues.

Regarding the quantitative analysis, the effect of MP versus MS was compared to evaluate whether one of the two methods was more effective in treating Class III growing patients. Since the meta-analysis could only be conducted for the outcomes reported in two or more studies, it was possible to compare the MP with MS outcomes only for some values, that is, for the sagittal parameters ANB, Wits, SNA, A-NPerp, Co-A, SNB, and Co-Gn, the vertical parameters PP-SN, Ar-Go-Me, and ANS-Me, and the dental parameters Overjet, Overbite, U1i-VRmx/Is-yMx/U1-VR, U1^PP/Is^PP/U1^NL/U1^MxP, U1^SN, and U6-VRmx/VRL-U6, but unfortunately, for no soft tissue parameters.

U1i-VRmx/ Is-yMx/ U1-VR, U1^PP/ Is^PP/ U1^NL/ U1^MxP, and U6-VRmx/ U6-VRL: these values refer to the same parameter, but they were given different names by different authors. The key clinical take-home messages derived from the meta-analysis are summarized in Table 4.

Sagittal parameters

ANB: A total of 12 comparisons provides information on this parameter. The overall effect estimate shows a mean ANB gain of 3.66°, with values of 3.05° in the MS group and 3.97° in the MP group. Therefore, the use of MS results in a mean ANB gain similar to that achieved with MP treatment (*p* = 0.403). The mean difference was estimated at 0.91°.

**Figure 4:**
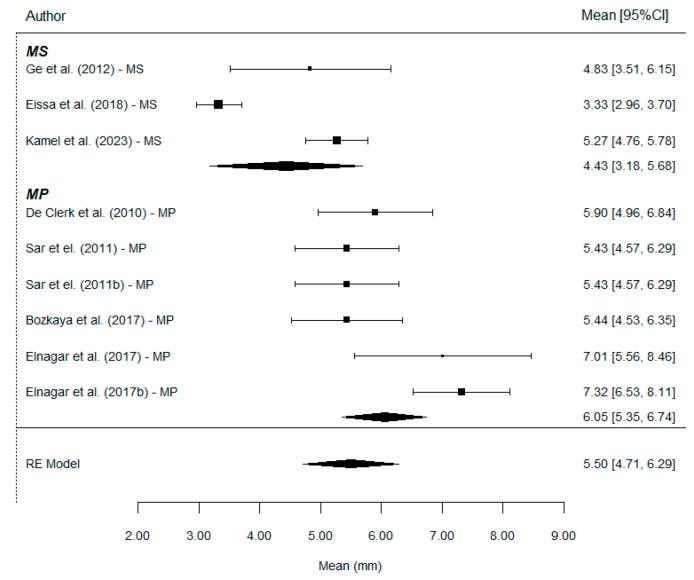
Forest plots of the difference for Wits values between MP and MS. CI, confidence interval [7,9,11,20,21,23,24].

WITS: A total of nine comparisons provides information on this parameter. The overall effect estimate shows a mean Wits increase of 5.50 mm, with values of 4.43 mm in the MS group and 6.05 mm in the MP group. Therefore, the use of MP results in a significantly greater Wits increase compared to MS treatment (*p* = 0.014). The mean difference was estimated at 1.64 mm (Figure 4).

**Figure 5:**
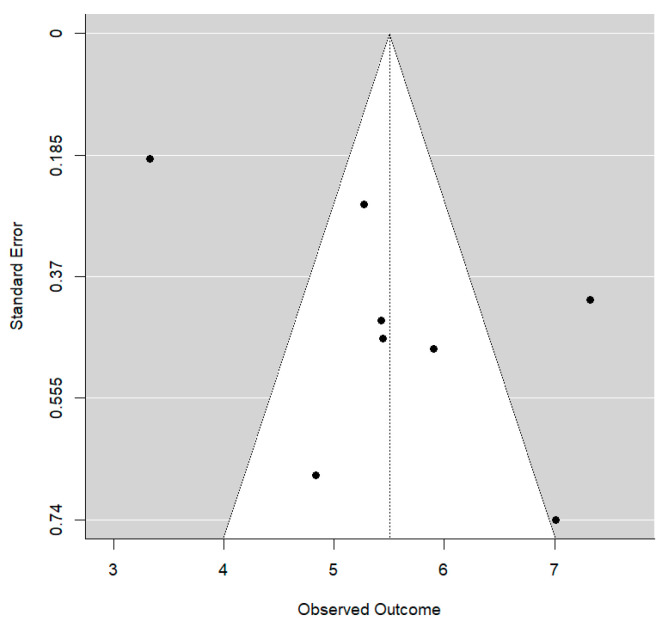
Funnel plot for Wits values.

In this case, the residual between-study variability after regression adjustment is high (I^2^ = 82.1%), although up to 43.3% of the initial total heterogeneity can be explained by the type of treatment. On the other hand, no publication bias was detected (*p* = 0.101). The Funnel plot shows clear asymmetry (Figure 5).

SNA: A total of 13 comparisons provides information on this parameter. The overall effect estimate shows a mean SNA increase of 2.85°, with values of 2.51° in the MS group and 3.01° in the MP group. There are no differences in SNA change between the two treatments (*p* = 0.559). The mean difference was estimated at only 0.5°.

A-Nperp: A total of six comparisons provides information on this parameter. The overall effect estimate shows a mean increase of 4.18 mm, with values of 4.45 mm in the MS group and 4.12 mm in the MP group. Both treatments result in a similar A-Nperp gain (*p* = 0.888). The mean difference was estimated at 0.22 mm.

Co-A: A total of seven comparisons provides information on this parameter. The overall effect estimate shows a mean increase of 4.76 mm, with values of 4.58 mm in the MS group and 4.81 mm in the MP group. Both treatments result in a similar change in Co-A (*p* = 0.825). The mean difference was estimated at 0.12 mm.

SNB: A total of 13 comparisons provides information on this parameter. The overall effect estimate shows a mean SNB reduction of −0.65°, with values of −0.53° in the MS group and −0.72° in the MP group. There are no differences in SNB change between the two treatments (*p* = 0.682). The mean difference was estimated at only 0.19°.

Co-Gn: A total of six comparisons provides information on this parameter. The overall effect estimate shows a mean increase of 1.85 mm, with values of 2.09 mm in the MS group and 1.76 mm in the MP group. Both treatments result in a similar change in Co-Gn (*p* = 0.662). The mean difference was estimated at 0.29 mm.

Vertical parameters

PP-SN: A total of seven comparisons provides information on this parameter. The overall effect estimate shows a mean decrease in PP-SN of −0.41°, with values of −0.46° in the MS group and −0.42° in the MP group. The mean difference in variation between the two groups was estimated at −0.02°. Evidently, such a small value does not represent a significant difference between the groups (*p* = 0.968).

Ar-Go-Me: A total of six comparisons provides information on this parameter. On average, the angle decreased by −1.86°, with values of −0.40° in the MS group and −2.59° in the MP group. The mean difference in variation between the two groups was estimated at 2.08°, without reaching statistical significance (*p* = 0.227).

Ans-Me: A total of eight comparisons provides information on this parameter. This dimension increased by 1.67 mm following the intervention: 0.66 mm in the MS group and 2.06 mm in the MP group. No significant treatment effect was observed (*p* = 0.149).

Dental parameters

Overjet: A total of 10 comparisons provides information on this parameter. The estimated mean overall advancement was +5.71 mm, with very similar partial results for both groups: 5.65 mm with MS and 5.76 mm with MP. No differences were found between treatments (*p* = 0.909).

**Figure 6:**
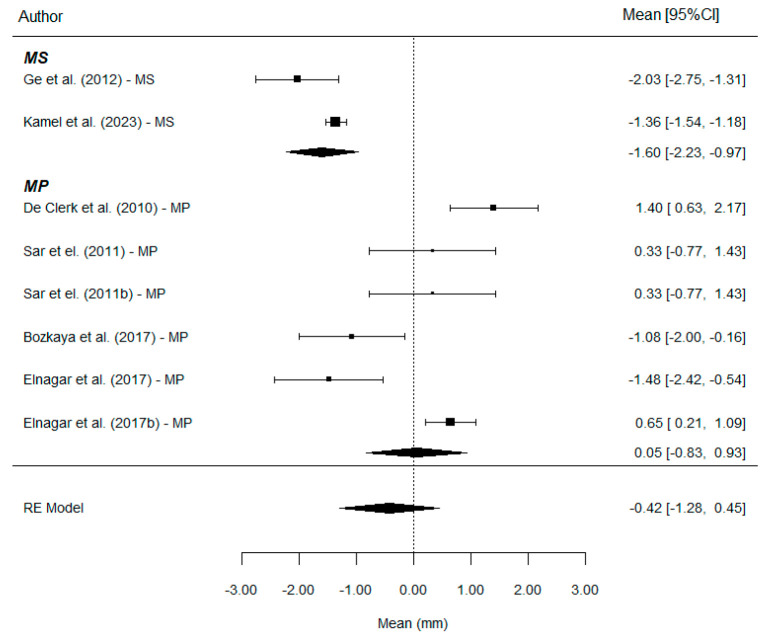
Forest plots of the difference for Overbite values between MP and MS. CI, confidence interval [7,9,11,20,21,24].

Overbite: A total of eight comparisons provides information on this parameter. The estimated mean reduction in overbite was −0.42 mm, with clearly different values between groups: −1.60 mm with MS and +0.05 mm with MP (Figure 6).

**Figure 7:**
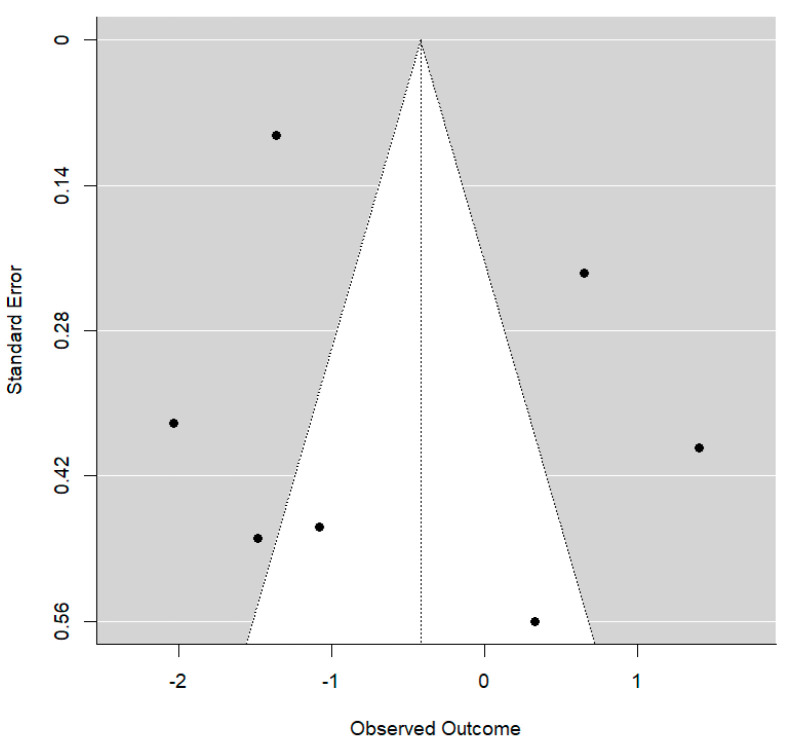
Funnel plot for overbite values.

With the use of MS, an additional mean overbite reduction of approximately 1.72 mm is achieved. This is a substantial effect size and reaches statistical significance (*p* = 0.029). The R^2^ value is consistent with a non-zero degree of explanation by the type of treatment, although a substantial amount of residual between-study variability remains. No publication bias was detected (*p* = 0.669): The Funnel plot shows clear asymmetry (Figure 7).

**Figure 8:**
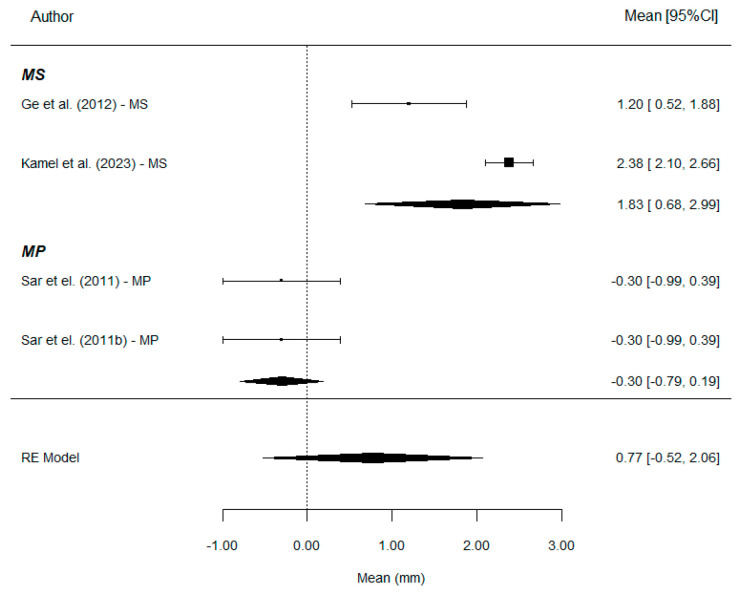
Forest plots of the difference for upper incisor protrusion values between MP and MS. CI, confidence interval [7,11,21].

Upper incisor protrusion (U1i-VRmx, Is-yMx, and U1-VR): A total of four comparisons provide information on this parameter. The mean variation across the four studies was estimated at 0.77 mm: 1.83 mm with MS and −0.30 mm with MP (Figure 8).

**Figure 9:**
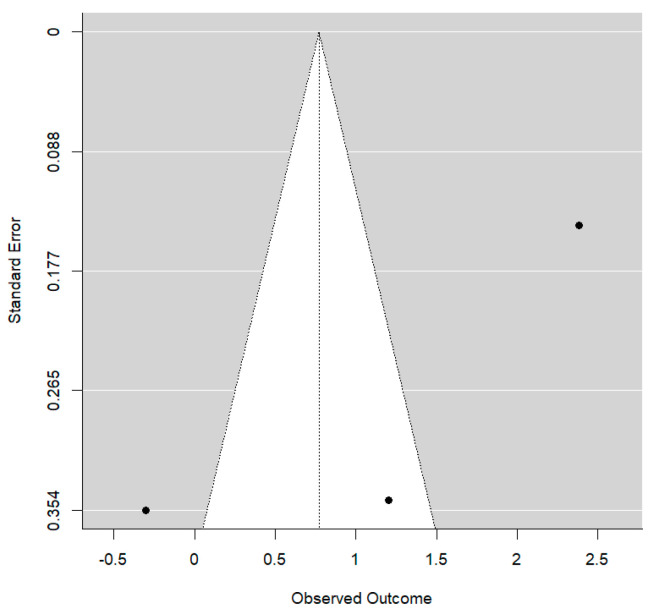
Funnel plot for upper incisor protrusion values.

Treatment with MS results in a significantly greater mean increase in this parameter compared to MP (*p* < 0.001). The mean difference between groups is substantial. This leads to a very high R^2^ estimate: 82% of the total uncertainty in the variation in the patient’s radiographic measurement is attributable to the type of treatment. However, the small number of included studies maintains resdual heterogeneity at moderately high levels (77%). Although the Egger’s test rejects the null hypothesis (*p* = 0.013), the result should be considered. The Funnel plot shows clear asymmetry (Figure 9).

Upper incisor proclination (U1^PP, Is^PP, U1^NL, and U1^MxP): A total of 10 comparisons provides information on this parameter. On average, the angle increased by 0.22°, with values of 0.37° in the MS group and 0.19° in the MP group. The mean difference in variation between the two groups was estimated at 0.14°, far from statistical significance (*p* = 0.927).

Upper incisor proclination (U1^SN): A total of four comparisons provides information on this parameter. On average, the angle increased by 2.04°, with values of 3.16° in the MS group and 0.91° in the MP group. The latter is the result of a weighted average of two extremely heterogeneous articles. The mean difference in variation between the two groups was estimated at 2.37°. This difference cannot be accepted as significantly different from zero (*p* = 0.563).

**Figure 10:**
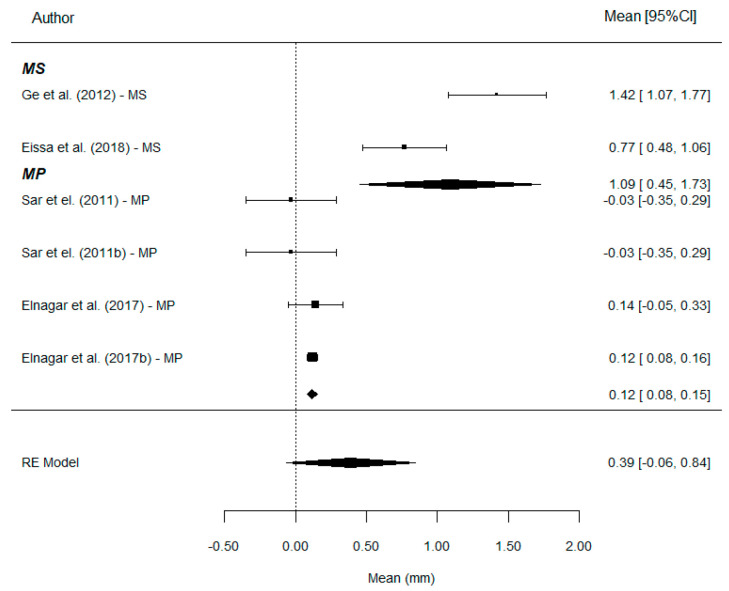
Forest plots of the difference for upper molar mesialization values between MP versus MS. CI, confidence interval [11,21,23,24].

Upper molar mesialization (U6-VRmx, U6-VRL): A total of six comparisons provides information on this parameter. The mean variation is positive, interpreted as an increase in length: +0.39 mm. With MS, +1.09 mm, and with MP, only +0.12 mm (Figure 10).

**Figure 11:**
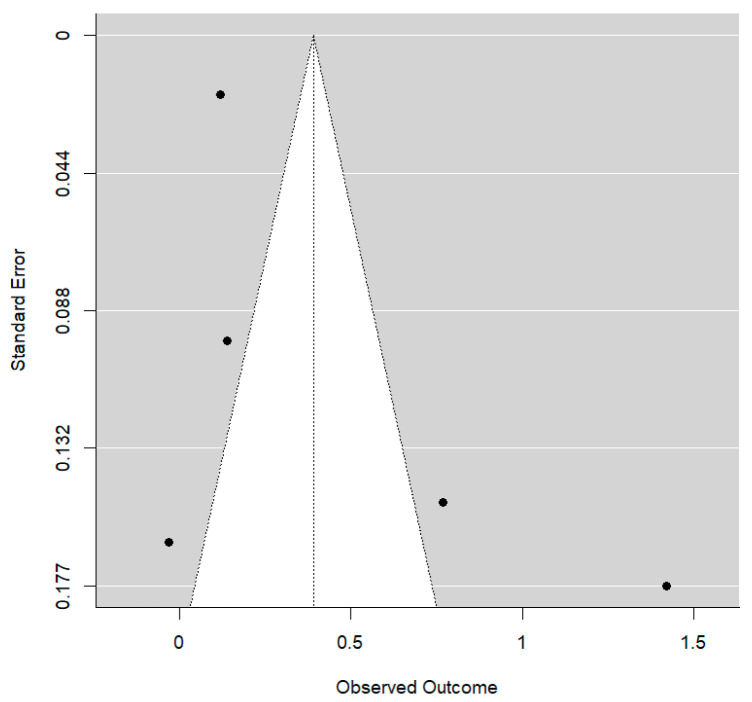
Funnel plot for upper molar mesialization values.

With the use of MS, a mean additional increase of 0.99 mm in this parameter is achieved. This is a significantly non-zero effect size (*p* < 0.001). In this model, the type of treatment strongly determines the level of variation (R^2^ = 92.5%). No publication bias was detected (*p* = 0.404). The Funnel plot shows clear asymmetry (Figure 11).

The Funnel plot analysis and Egger’s test did not reveal statistically significant results, suggesting a likely absence of publication bias, namely the tendency to preferentially publish studies with positive or significant outcomes.

## 4. Discussion

The correction of skeletal Class III malocclusion during growth is of paramount importance to improve facial esthetics, restore proper occlusal function, and, when possible, reduce or even eliminate the need for orthognathic surgery in adulthood. Early orthopedic intervention aims to stimulate forward growth of the maxilla and harmonize the maxillo–mandibular relationship before sutural maturation limits the orthopedic response, thereby enhancing long-term stability of the results [27].

Traditionally, maxillary protraction has been carried out using tooth-anchored facemask therapy, which has been shown to produce clinically significant forward movement of the maxilla in growing patients [28,29]. However, conventional FM protocols transmit orthopedic forces through the dentition, which may lead to undesirable dentoalveolar compensations such as mesialization and extrusion of maxillary molars, proclination of upper incisors, and clockwise mandibular rotation, reducing the net skeletal gain [30,31].

To overcome these limitations, bone-anchored maxillary protraction (BAMP) techniques have been developed. By transferring orthopedic forces directly to the skeletal structures through miniscrews (MSs) or miniplates (MPs), BAMP aims to maximize skeletal effects while minimizing unwanted dental movements [9]. Miniscrews are typically connected to a palatal bone-borne or hybrid expanders equipped with hooks for connecting elastics to a facemask [32], or, less frequently, to a mandibular anchorage featuring intraoral hooks [7]. Miniscrews can also be placed in the zygomatic buttress of the maxilla, which results a substantial region where skeletal anchorage can be placed [21]. Miniplates, on the other hand, are usually employed in two ways: the first method involves miniplates positioned in the infrazygomatic crest and in the anterior region of the mandible, connected by intermaxillary elastics [9]; the second way provides miniplates screwed into the anterior region of the maxilla, connected to the facemask using elastics [10].

Nonetheless, the adoption of skeletal anchorage is not without drawbacks. Reported complications include mobility or loss of the anchorage device, soft tissue irritation, and, in the case of miniplates, the need for flap surgery with increased invasiveness and associated morbidity [9,33,34]. Additionally, in some cases, treatment initiation may be delayed until the eruption of lower canines to allow safe placement of mandibular miniplates or bars, which could reduce the orthopedic potential due to advancing skeletal maturation [35,36].

The aim of this study was to provide a comparison between miniplate and miniscrew anchorage systems in the orthopedic treatment of growing patients affected by Class III malocclusion, evaluating whether any differences exist between these two BAMP approaches. Only prospective RCT and n-RCT studies were included in this study. The search criteria had no limitations about languages, year, and type of publication, investigating five databases (PubMed, Cochrane, Embase, Scopus, and Web of Science), and, additionally, a manual search strategy. To minimize potential bias in study selection and data extraction, two independent reviewers were involved in both the screening process and the assessment of risk of bias and evidence quality.

In this systematic review, we selected seven studies using miniplates and four studies using a miniscrew-anchored system, and, in particular, three studies used MP with intermaxillary elastics [9,22,26], four studies used MP with FM [10,11,20,24], one study used MS with intermaxillary elastics [7], two studies used MS with FM [21,25], and one study used MS with inverted forsus [23].

Given that we found only one study in the literature that directly compares MS and MP anchorage [37], we included articles based on our inclusion and exclusion criteria that compared MS versus control and MP versus control and extracted all outcomes that were statistically significant. A meta-analysis was then conducted to assess whether these results showed different significance with MS or MP anchorage, comparing the outcomes.

The two groups (MS and MP) presented with similar characteristics at baseline in terms of sagittal parameters (e.g., ANB ranging from −1° to −2.5° and WITS from −4 mm to −6 mm), vertical parameters (e.g., FMA 25.2–27.5° and SN-GoGn 32.5–34.2°), dental parameters (e.g., U1-SN from 102.7 to 107.1° and IMPA from 83.5 to 89.4°), and soft tissue parameters (e.g., nasolabial angle from 100.5 to 104.6° and upper lip to E-line from −3.1 to −3.8 mm). Participant numbers and mean age were also comparable across groups. This suggests relative baseline homogeneity in malocclusion severity across the studies, allowing for valid direct comparisons in the meta-analysis.

The qualitative analysis of our review confirmed that the studies included in this research consistently report greater skeletal sagittal effects and fewer dental side effects with skeletal anchorage compared to traditional tooth-borne anchorage [10,11,21,25], suggesting that skeletal anchorage can enhance the orthopedic response and potentially reduce the magnitude of surgical correction required in non-growing patients.

These findings are consistent with previous systematic reviews by Cornelis et al. [13] and Wang et al. [14], which reported more pronounced orthopedic effects with skeletal anchorage in maxillary protraction. However, they contrast with Rutili et al. [12], who found only a modest, statistically significant increase in the SNA angle (mean difference of 0.8°) when comparing facemask therapy with skeletal versus dental anchorage, questioning the true clinical relevance of such a difference. Rutili et al. [12], however, agreed that skeletal anchorage systems reduce dental anchorage loss, minimizing upper incisor proclination and mesial migration of maxillary molars.

Regarding the values of the vertical parameters, there is disagreement among the authors; some have found a reduction in the vertical dimension [9,10,11], while others found an increase in it [10,20]. Sometimes the same study has evaluated an increase in verticality for some values and a decrease in others [7,11,24]. Soft tissue improvements were consistent in both MS and MP groups compared to controls, especially with increased upper lip protrusion and chin retrusion [7,9,11,20,23].

However, our primary objective was to compare MP- and MS-anchored systems, highlighting any differences through a quantitative analysis. The comparison between the two skeletal anchorage types showed that the MP group achieved greater improvement in the maxillo–mandibular relationship, particularly in the Wits appraisal. The use of miniplates, often combined with intermaxillary elastics or facemasks directly anchored to the bone (via hooks), involves no dental anchorage, unlike many MS-based systems. This exclusive application of force to the bone may explain the superior correction of the maxillo–mandibular relationship observed, aligning with Wang et al. [14], who suggested that “bone anchorage with intermaxillary protraction” may be the most effective method to correct Class III intermaxillary discrepancies.

Cornelis et al. [13] assessed that skeletal anchorage protocols, especially those using bilateral miniplates with intermaxillary elastics (BAC3Es), could result in a more pronounced skeletal effect and minimal dental compensation. Similarly, Miranda et al. [37] also found more favorable clinical effects in maxillary advancement with miniplates than with miniscrews. He also suggested that the observed differences may be due to the MS group using hybrid anchorage in the maxilla, while the MP group employed a fully skeletal anchorage approach.

On the other hand, the MS group experienced more unwanted dentoalveolar effects, such as reduced overbite, upper incisor proclination, and mesialization of maxillary molars. This finding has important clinical implications for patients presenting with significant upper incisor protrusion at baseline, a common compensatory mechanism in Class III skeletal malocclusion. This suggests that hybrid skeletal anchorage systems, typical of MS protocols, dissipate part of the orthopedic forces through the dentition, leading to undesired dentoalveolar compensations during Class III correction. This is consistent with Miranda’s study, where the MS group demonstrated greater dental side effects, including mesial migration of maxillary first molars and labial inclination of upper incisors [37].

Given that skeletal anchorage was introduced to enhance orthopedic effects while minimizing dental side effects, our meta-analysis suggests that miniplates are more effective than miniscrews in achieving this goal. However, the surgical invasiveness associated with miniplate placement should be considered.

The comparison between the two BAMP approaches did not reveal any significant difference regarding the vertical skeletal values between MP and MS, consistent with the findings of Miranda et al., who also reported similar vertical changes in the maxilla and mandible between groups. However, regarding angular changes, they observed a greater counterclockwise rotation of both the palatal and mandibular planes in the MP group compared to the MS group [37].

Our meta-analysis indicates that miniplates tend to produce a more pronounced improvement in the intermaxillary relationship compared to miniscrews, which, in contrast, are associated with more undesirable dentoalveolar side effects.

From a clinical perspective, MPs offer several advantages: they provide a stronger orthopedic effect on the intermaxillary relationship, rely on purely skeletal biomechanics since elastics are attached directly to bone-anchored plates, and are particularly valuable when the objective is to avoid additional proclination or mesialization of molars. However, these benefits come with important limitations, including surgical invasiveness (flap procedures for placement and removal) and the related morbidity [33], timing constraints (as mandibular placement often requires eruption of the lower canines, potentially reducing orthopedic effectiveness if therapy is delayed), and the need for a surgical environment and specialized expertise. MPs should therefore be considered for Class III patients with significant dental compensation (such as proclined upper incisors and reduced overbite), where minimizing dental side effects is crucial.

On the other hand, MSs also present some advantages: they are less invasive and easier to place, offer versatile anchorage sites (including the palate with bone-borne or hybrid expanders, mandibular symphysis, and infrazygomatic crest), and generally show good patient acceptability [38,39]. Nevertheless, their main drawbacks include greater dental side effects (upper incisor proclination, overbite reduction, and mesialization of upper molars), most likely related to the hybrid nature of some devices, which can lead to force dispersion.

In clinical practice, MSs may be preferred in Class III patients where flap surgery is contraindicated or should be avoided, when anatomical or age-related factors prevent safe placement of MPs, or in cases with retruded upper incisors and increased overbite, where minor dental side effects are clinically tolerable.

The risk of bias assessment showed some concerns in most RCTs and serious to critical risk in several nRCTs, mainly due to confounding and deviations from intended interventions. These limitations may reduce the reliability of pooled estimates; however, the GRADE ratings (moderate to high certainty) support that the main conclusions remain reasonably robust.

As a final point, the present study has highlighted the following limitations.

There is a limited number of RCTs and nRCts evaluating the effects of treatments with miniscrews and miniplates.

There is a heterogeneity of orthodontic treatment protocols used across studies, including variations in BAMP strategies.

There is a lack of direct comparison studies between MS and MP anchorage.

Furthermore, most of the available studies have a low or moderate level of evidence, and the number of high-quality randomized controlled trials (RCTs) is still limited.

## 5. Conclusions

Both MS and MP skeletal anchorage systems show significant improvements compared to traditional dental anchorage or untreated patients. Skeletal anchorage results in improvements in maxillary advancement and the maxillo–mandibular relationship, better vertical skeletal control, fewer unwanted dental effects, and a less convex profile with upper lip protrusion and retrusion of the soft tissues in the chin area.

The comparison between the values obtained with MP and MS yielded the following results:

1. Skeletal Sagittal Outcomes: It appears that the use of MP leads to improvements in the intermaxillary relationship compared to MS.
2. Skeletal Vertical Outcomes: No differences between the groups.
3. Dental effects: Treatment with MS—likely due to the hybrid nature of most miniscrew-based systems—seems to have more undesirable dentoalveolar effects, particularly a greater reduction in overbite, increased protrusion of the upper incisors, and forward movement of the upper molars.
4. Soft tissue: Unfortunately, the data collected were insufficient to perform a meta-analysis.

Our meta-analysis outcomes should be interpreted with caution due to the limited quality and quantity of the available evidence and the heterogeneity among studies.

Future research is essential: well-designed, high-quality randomized controlled trials (RCTs) that directly compare the effects of MSs and MPs with larger sample sizes and standardized outcome measures are urgently needed. Long-term follow-up studies are especially important to determine the stability and durability of treatment outcomes, to better differentiate between MS and MP effects, and to clarify soft tissue adaptations.

## Figures and Tables

**Table 1:** Characteristics of included studies.

Authors, Year, Country	Type of Study	Sample Group Size	Control Group Size	Sample Characteristics (Age, Gender)	Control Characteristics (Age, Gender)	Intervention (Screw or Plate)	Control Group Type	Treatment Duration
De Clerk et al. (2010) [9] US and Italy	nRCT	21	18	9.0–13.6 M: 11, F: 10	8.8–13.1 M: 11, F: 7	MP	Untreated	≈1 year
Sar et al. (2011) [11] Turkey	nRCT	30	30	10.9 M: 20, F: 10	10.7 ± 1.3 M: 15, F: 15	MP	Tooth-borne Untreated	6.78 mo (SG) 9.45 mo (CG)
Ge et al. (2012) [21] China	RCT	20	23	10.4 M: 9, F: 11	10.6 M: 11, F: 12	MS	Tooth-borne	11 mo (SG) 13 mo (CG)
Eid et al. (2016) [22] Egypt	nRCT	10	10	9–12 M: 5, F: 5	9–12 M: 7, F: 3	MP	Untreated	1 year
Bozkaya et al. (2017) [20] Turkey	nRCT	18	18	11.4 ± 1.28 M: 8, F: 10	10.6 ± 1.12 M: 9, F: 9	MP	Untreated	1.08 ± 0.35 y (SG) 0.82 ± 0.28 y (CG)
Elnagar et al. (2017) [24] Egypt and US	RCT	20	10	10–14 M: 13, F: 7	10–14 M: 7, F: 3	MP	Untreated	8–8.9 mo (SG) 9.4 mo (CG)
Eissa et al. (2018) [23] Egypt	nRCT	16	16	12.45 ± 0.87 M: 7, F: 9	11.95 ± 1.04 M: 8, F: 8	MS	Untreated	6.4 ± 1.46 mo
Seiryu et al. (2020) [25] Japan	RCT	19	20	11.1 ± 1.3 M: 12, F: 7	10.5 ± 1.8 M: 12, F: 8	MS	Tooth-borne	1.9 year ± 9.9 mo (SG) 1. 9 year ± 10.2 mo (CG)
Liang et al. (2021) [10] China	RCT	20	21	10.7 ± 1.3 M: 8, F: 12	10.5 ± 1.1 M: 11, F: 10	MP	Tooth-borne	10.6 mo (SG) 12.1 mo (CG)
Kamel et al. (2023) [7] Egypt	RCT	17	13	11.3 ± 1.1 M: 9, F: 8	11.5 ± 1.2 M: 8, F: 5	MS	Untreated	11.9 mo
Mandall et al. (2024) [26] UK	RCT	28	29	12.9 ± 0.7 M: 17, F: 11	12.6 ± 0.9 M: 19, F: 10	MP	Untreated	≈18 mo

**Table 2:** Significant values and their variation for studies involving the miniplate-treated group.

Authors, Year, Type of Control Group	Significant Sagittal Skeletal Differences (MP vs. C) *p* < 0.05	Significant Vertical Skeletal Differences (MP vs. C) *p* < 0.05	Significant Dental Differences (MP vs. C) *p* < 0.05	Significant Soft Tissues Differences (MP vs. C) *p* < 0.05
**De Clerk et al. (2010)** [9]**,** **untreated**	Maxillary advancement: increase in SG Maxillary length: increase in SG Maxillary–mandibular relationship: improvement increase in SG Mandibular posterior relocation: increase in SG Mandibular length: increase in CG	Mandibular divergence: decrease in SG Clockwise rotation of palatal plane: increase in SG	Lower incisor proclination: increase in SG Overjet: increase in SG Overbite: increase in SG	Upper lip protrusion: increase in SG Lower lip retrusion: increase in SG Chin retrusion: increase in SG
**Sar et al. (2011)** [11]**,** **tooth borne**	Maxillary advancement: increase in SG Mandibular posterior relocation: decrease in SG Maxillary–mandibular relationship improvement: increase in SG Mandibular length: increase in CG	Posterior rotation of the mandible: increase in CG Anterior facial height: increase in CG Total facial height: increase in CG Anterior rotation of the maxilla: increase in CG	Upper incisor protrusion: increase in CG Upper incisor proclination: increase in CG Lower incisor retrusion: increase in SG Lower incisor retroclination: increase in SG Upper molar mesialization: increase in CG	Upper lip protrusion: increase in SG Soft tissue total facial height: increase in CG Soft tissue lower facial height: increase in CG
**Sar et al. (2011)** [11]**,** **untreated**	Maxillary advancement: increase in SG Mandibular posterior relocation: decrease in SG Maxillary–mandibular relationship improvement: increase in SG Mandibular length: increase in CG	Posterior rotation of the mandible: increase in SG Anterior rotation of the maxilla: increase in SG Anterior facial height: increase in SG Total facial height: increase in SG	Upper incisor protrusion: increase in CG Upper incisors proclination: increase in CG Lower incisor retrusion: increase in SG Lower incisor retroclination: increase in SG Overjet: increase in SG	Upper lip protrusion: increase in SG Soft tissue total facial height: increase in SG Soft tissue lower facial height: increase in SG Chin retrusion: decrease in SG Lower lip retrusion: increase in SG
**Eid et al. (2016)** [22]**,** **untreated**	Maxillary advancement: increase in SG Maxillary–mandibular relationship improvement: increase in SG Mandibular posterior relocation: increase in SG	None	Upper incisor proclination: increase in SG Upper incisor protrusion: increase in SG	NR
**Bozkaya et al. (2017)** [20]**,** **untreated**	Maxillary advancement: increase in SG Mandibular posterior relocation: increase in SG Maxillary–mandibular relationship improvement: increase in SG	Posterior rotation of the mandible: increase in SG Clockwise rotation of the maxilla: increase in SG Anterior face height: increase in SG	Lower incisor retrusion: increase in SG Lower incisor retroclination: increase in SG Upper molar extrusion: increase in SG Overjet: increase in SG Overbite: decrease in SG	Upper lip protrusion: increase in SG Lower lip retrusion: increase in SG
**Elnagar et al. (2017)** [24]**, untreated**	Maxillary advancement: increase in SG Maxillary length: increase in SG Mandibular posterior relocation: increase in SG Maxillary–mandibular relationship improvement: increase in SG	Posterior rotation of the mandible: increase in SG Gonial angle: decrease in SG	Overjet: increase in SG Overbite: decrease in SG IMPA: decrease in SG	Upper lip protrusion: increase in SG Lower lip retrusion: increase in SG Chin retrusion: increase in SG
**Elnagar et al. (2017)** [24]**, untreated**	Maxillary advancement: increase in SG Maxillary length: increase in SG Mandibular posterior relocation: increase in SG Maxillary–mandibular relationship improvement: increase in SG	Gonial angle: decrease in SG	Overjet: increase in SG Overbite: decrease in SG	Upper lip protrusion: increase in SG Chin retrusion: increase in SG
**Liang et al. (2021)** [10]**,** **tooth borne**	Maxillary advancement: increase in SG Maxillary length: increase in SG	Clockwise rotation of palatal plane: increase in SG	Lower incisor retroclination: decrease in SG Lower incisor retrusion: decrease in SG Upper molar extrusion: decrease in SG	NR
**Mandall et al. (2024)** [26]**,** **untreated**	Maxillary–mandibular relationship: increase in SG	None	Overjet: increase in SG	NR

**Table 3:** Significant values and their variation for studies involving the miniscrew-treated group.

**Authors, Year, Type of** **Control Group**	**Significant Sagittal** **Skeletal Differences (MS vs. C) *p* < 0.05**	**Significant Vertical Skeletal Differences (MS vs. C) *p* < 0.05**	**Significant Dental** **Differences (MS vs. C) *p* < 0.05**	**Significant Soft** **Tissues Differences (MS vs. C) *p* < 0.05**
**Ge et al. (2012)** [21]**,** **tooth borne**	None	None	Upper incisor proclination: increase in CG Upper incisor protrusion: increase in CG Upper molar mesialization: increase in CG	None
**Eissa et al. (2018)** [23]**,** **untreated**	Maxillary advancement: increase in SG Maxillary–mandibular relationship improvement: increase in SG	Counterclockwise rotation of occlusal plane: increase in SG	Upper incisor proclination: increase in SG Upper incisor protrusion: increase in SG Upper incisor intrusion: increase in SG Lower incisor retroclination: increase in SG Lower incisor retrusion: increase in SG Upper molar mesialization: increase in SG Lower molar distalization: increase in SG Lower molar intrusion: increase in SG Overjet: increase in SG	Upper lip protrusion: increase in SG Lower lip retrusion: increase in SG Nasolabial angle: decrease in SG
**Seiryu et al. (2020)** [25]**,** **tooth borne**	Maxillary advancement: increase in SG Maxillary–mandibular relationship improvement: increase in SG	None	Upper incisor proclination: increase in CT	NR
**Kamel et al. (2023)** [7]**,** **untreated**	Maxillary advancement: increase in SG Mandibular posterior relocation: increase in SG Maxillary–mandibular relationship improvement: increase in SG Maxillary length: increase in SG	Mandibular divergence: decrease in SG Anterior face height: increase in SG	Upper incisor protrusion: increase in SG Upper incisor proclination: increase in SG Upper molar mesialization: increase in SG Upper molar extrusion: increase in SG Lower incisor extrusion: decrease in SG Lower incisor protrusion: increase in SG Overjet: increase in SG Overbite: decrease in SG	Upper lip protrusion: increase in SG Lower lip retrusion: increase in SG Chin retrusion: increase in SG

**Table 4:** Key clinical take-home messages derived from meta-analysis outcomes.

Outcome	MS (Miniscrews)	MP (Miniplates)	Clinical Message
**ANB angle**	+3.05°	+3.97°	Both improve sagittal skeletal relationship; no significant difference
**WITS appraisal**	+4.43 mm	+6.05 mm	MP achieve greater improvement in intermaxillary relationship
**SNA**	+2.51°	+3.01°	Both advance the maxilla; no significant difference
**A-NPerp**	+4.45 mm	+4.12 mm	Both improve A-point position; no significant difference
**Co-A (maxillary length)**	+4.58 mm	+4.81 mm	Comparable maxillary length increase; no significant difference
**SNB**	–0.53°	–0.72°	Both induce mandibular retrusion; no significant difference
**Co-Gn (mandibular length)**	+2.09 mm	+1.76 mm	Both increase mandibular length; no significant difference
**PP-SN**	–0.46°	–0.42°	Both show minimal palatal plane rotation; no significant difference
**Ar-Go-Me**	–0.40°	–2.59°	Both reduce gonial angle; no significant difference
**ANS-Me**	+0.66 mm	+2.06 mm	Both increase lower anterior facial height; no significant difference
**Overjet**	+5.65 mm	+5.76 mm	Similar overjet improvement; no significant difference
**Overbite**	–1.6 mm	+0.05 mm	MS causes greater overbite reduction
**Upper incisor protrusion**	+1.83 mm	–0.30 mm	MS increases upper incisor protrusion
**Upper incisor proclination**	≈+3°	≈+1°	Both increase upper incisor proclination; no significant difference
**Upper molar mesialization**	+1.09 mm	+0.12 mm	MS causes greater molar mesialization

## Data Availability

The data presented in this study are available in the article.

## Supplementary Materials

The following supporting information can be downloaded at: https://www.mdpi.com/article/10.3390/bioengineering12101065/s1, Table S1: search strategy for each database; Table S2: description of PICOs of this systematic review; Table S3: Grading of Recommendation, Assessment, Development, and Evaluation (GRADE) analysis for the studies about treatments with miniplates; Table S4: Grading of Recommendation, Assessment, Development, and Evaluation (GRADE) analysis for the studies about treatments with miniscrews.

## Abbreviations

The following abbreviations are used in this manuscript:

MSs: Miniscrews, Cs: Controls, NR: Not reported, SG: Sample group, CG: Control group, M: male, F: female, RCTs: randomized clinical trials, nRCTs: non-randomized clinical trials, BAMP: Bone-anchored maxillary protraction, FM: Tooth-anchored face mask therapy, mo: Months, A-VR: Perpendicular distance from A to VR, A-NPerp: Perpendicular distance from A-point to Nasion Perpendicular line to FH plane, SNA: Angle between the anterior cranial base and (Nasion-point A) line, B-VR and B-VerT: Perpendicular distance from B to Vertical T, Pg-VR and Pg-VerT: Perpendicular distance from Pogonion to Vertical T, SNB: Angle between the anterior cranial base and (Nasion-point B) line, ANB: Angle between (Nasion-point A) and (Nasion-point B) lines, SNA minus SNB- Wits: Distance between AO and BO projections of points A and B to the Occlusal Plane, Co-A: the distance between Condylion and point A, ANS-V plane: Distance from ANS point to the V plane, A-V plane: Distance from A point to the V plane, PNS-ANS: Distance from the PNS point to the ANS point, Cd-A: Distance between the condylar point (Cd) and point A, Mx-Md diff: Maxillo–mandibular differential, N-A-Pog: Angle between point N-A-Pog, Ptm-VertT and Ptm-VR: Perpendicular distance from Ptm-point to Vertical T, Or-VertT and Or-VR: Perpendicular distance from Or-point to Vertical T, Co-Gn: Linear distance from Gnathion (Gn) to Condylion (Co), SNO°: Sella-Nasion-Orbitale angle, SN-ANS°: Angle between Sella, Nasion and Anterior Nasal Spine, A-y: Perpendicular distance from A-point to the perpendicular plane to the T-W plane at the T point, B-y: Perpendicular distance from B-point to the perpendicular plane to the T-W plane at the T point, A-VerT: projection of point A on Vertical T, ANS-PNS/GoGn: Angle between ANS-PNS plane and mandibular plane (GoGn), NPerp–Pg : Horizontal distance from pogonion to a line perpendicular to the Frankfort horizontal plane passing through Nasion, (A–VR)—(B–VR): Difference between the horizontal positions of Point A and Point B relative to the vertical reference plane, Cd-Gn: Distance from Condylion to Gnathion, li-xMd: Vertical distance from the lower incisor to mandibular plane, Ar-Go-Me : Gonial angle between the (Articulare-Gonion) line and (Gonion-Menton) line, PP-SN: Angle between the PP plane and the SN plane, SN^GoGn: Angle between the Sella–Nasion (SN) plane and the GoGn mandibular plane, HR^GoMe: Angle between the HR point (temporomandibular joint) and Gonion (Go), ANS-Me: Distance between the Anterior Nasal Spine and Me, N-Me: Distance between the Nasion and Menton, ML-SBL: Angle between the mandibular plane and the cranial base, Co-Go-Me: Gonial angle formed by Condylion (Co), Gonion (Go), and Menton (Me), NL-ML: Angle between the palatine plane (NL) and the mandibular plane (ML), SN^Occ: angle between the Sella–Nasion (SN) plane and the occlusal plane, A-x: Perpendicular distance from A-point to the tuberculum sella-wing (T-W) plane, B-x: Perpendicular distance from B-point to the tuberculum sella-wing (T-W) plane, Co-x: Perpendicular distance from Co-point to the tuberculum sella-wing (T-W) plane, HR-PNS: Horizontal distance from the Posterior Nasal Spine to the horizontal reference plane, HR^PP: Angle between the palatal plane and the horizontal reference plane, BaNa^PtGn: Angle between the cranial base and the mandibular plane, Mandibular rotation: Rotation of the mandible measured via structural superimposition, , S–Go/N–Me: Ratio of the posterior facial height (sella–gonion) to the total anterior facial height (Nasion–Menton), Is-yMx: Perpendicular distance from the maxillary incisor tip to yMx, Is-PP: Angle between the long axis of the maxillary incisor and maxillary plane, Ms-yMx: Perpendicular distance from the mesio-buccal cusp tip of the maxillary first permanent molar to yMx, Ms-xMx: Perpendicular distance from mesio-buccal cusp tip of the maxillary first permanent molar to xMx, Ii-yMd: Perpendicular distance from the mandibular incisor tip to yMd, Ii-MP: Angle between the long axis of the mandibular incisor and the mandibular plane (Go-Gn), U6-FH: Distance from the U6 point to the FH plane, U6-PP: Distance from the U6 point to the PP plane, U1-VR: Perpendicular distance from the upper incisor tip to the maxillary vertical reference plane (VR) and a vertical line passing through PNS and perpendicular to maxillary horizontal reference plane (drawn along the ANS-PNS line), U1i-VRmx: Distance from the upper incisor (U1i) to a vertical reference line drawn through the maxilla (VRmx), U6-VR: Perpendicular distance from the mesio-buccal cusp tip of the maxillary first permanent molar to the maxillary vertical reference plane (VR) and a vertical line passing through PNS and perpendicular to maxillary horizontal reference plane (drawn along the ANS-PNS line), U6-VRmx: Distance from upper molars (U1) to a vertical reference line drawn through the maxilla (VRmx), U1^HRmx: Angle between the long axis of the upper incisor and a horizontal reference line through the maxilla (HRmx), U1^PP: Angle between the upper incisor and the palatal plane, L1i–VRmd: Distance from the lower incisor and VRmx, L1^ML: Angle between the lower incisor and the mandibular plane (Go-Me), U1^SN: Angle between the long axis of upper incisor (U1) and the Sella–Nasion plane (SN), U1^L1: Inter-incisors’ angle, U1-NPog: distance between the upper incisor (U1) and the NPog line, U1^NA: Angle between the long axis of the upper incisor (U1) and the Nasion-A point line (NA), U1-NA: perpendicular distance from the upper incisor tip to the NA line, HRL-U1: perpendicular distance from the upper incisor tip to the horizontal reference line (HRL), L1^NB: Angle between the long axis of lower incisor (L1) and the Nasion-B point line (NB), L1-NB: perpendicular distance from the lower incisor tip to Nasion-B point line (NB), VRL-U6: Perpendicular distance from the upper molar to vertical reference line (VRL), VRL-L6: Perpendicular distance from the lower molar to vertical reference line (VRL), HRL-L6: Perpendicular distance from the lower molar to the horizontal reference line (HRL), L1-y2: Perpendicular distance from the lower incisor tip to the perpendicular plane to the Go-Gn plane at the Go point, U6-A pt: Distance between the most distal point of the upper first molar (U6) and the A-Pt plane, L1-GoMe: Acute angle between the long axis of the mandibular (lower) central incisor (l1) and the mandibular plane defined by the Gonion–Menton (go-Me) line, L1^GoGn: Angle between the long axis of lower incisor (L1) and the mandibular plane, U6-x1: Vertical displacement of the upper molar (U6) along the X-axis, A’-VR: Perpendicular distance from A’ to VR, Ls-VR: Perpendicular distance from labrale superius to VR, Li-VR and Llilp-VertT: Perpendicular distance from the lower lip to VR, B’-VertT: Perpendicular distance from B’ to VR, Pg’-VR and Pg’-VertT: Perpendicular distance from soft tissue Pogonion to VR, UL-VR and Ulip-VertT : Perpendicular distance from the upper lip to VR, Sn-VertT: Perpendicular distance from the subnasal to VR, UL-E plane: Perpendicular distance from the upper lip to the E-plane (Prn-Pog’), LL-E plane: Perpendicular distance from the lower lip to the E-plane (Prn-Pog’), NLA: Nasolabial angle, U6-VR: Perpendicular distance from U6 to VR, UL-S line: Perpendicular distance from the upper lip to Steiner’s line, LL-S line: Perpendicular distance from the lower lip to Steiner’s line, N’-Me’: Soft tissue facial height from soft tissue Nasion to soft tissue Menton, Sn-Me’: Soft tissue lower facial height from the subnasal to soft tissue Menton, LL–VR: horizontal distance from the lower lip to the vertical reference plane.
