# Supplementary material for: Comparative Evaluation of the Effects of Miniscrew and Miniplate Skeletal Anchorage in the Orthopedic Treatment of Growing Class III Malocclusion: A Systematic Review and Meta-Analysis
Source: Bioengineering (Basel). 2025 Sep 30;12(10):1065. doi: 10.3390/bioengineering12101065 (PMC12561213; doi:10.3390/bioengineering12101065)
Supplement: Supplementary file 1 [file bioengineering-12-01065-s001.zip › bioengineering-3854780-supplementary.pdf]

bioengineering

Supplementary Materials

Systematic Review

Comparative Evaluation of the Effects of Mini-Screw and Mini-Plate Skeletal Anchorage in the Orthopedic Treatment of Growing Class III Malocclusion: A Systematic Review and Meta-Analysis

Giuliano Irlandese[1], Giulia Perrotta[1], Vittoria Marsili[1], Laura Carboni[1], Alessio Verdecchia[1,2]*, Enrico Spinas[1]*

[1] Department of Surgical Sciences, Postgraduate School in Orthodontics, University of Cagliari, 09124 Cagliari, Italy; giuliano.irlandese@gmail.com (G.I.), drgiuliaperrotta@gmail.com (G.P.), vittoriamarsili15@gmail.com (V.M.) lracarbons@gmail.com (L.R.), verdecchia.belli.a@gmail.com (A.V.), enricospinas@tiscali.it (E.S.)

[2] Orthodontics Division, Instituto Asturiano de Odontología, Universidad de Oviedo, 33006 Oviedo, Spain; verdecchia.belli.a@gmail.com (A.V.)

* Correspondence: verdecchia.belli.a@gmail.com (A.V.); enricospinas@tiscali.it (E.S.)

Table S1. Search strategy for each database.

Database	Search Strategy
**Pubmed**	(("maxillary advancement") OR ("maxillary protraction")) OR ("skeletal class III malocclusion")) AND ("miniscrew-assisted")) OR ("miniscrew-anchored")) OR ("orthodontic miniscrews")) OR ("orthodontic miniplates")
**Web Of Science**	((ALL=("maxillary protraction")) OR ALL=(“maxillary advancement”)) OR ALL=(“skeletal class III malocclusion”)) AND ALL=(“miniscrew-assisted”)) OR ALL=(“miniscrew-anchored”)) OR ALL=(“orthodontic miniplates”)) OR ALL=(“orthodontic miniscrews”)
**Cochrane**	("maxillary protraction"):ti,ab,kw OR ("skeletal class III malocclusion"):ti,ab,kw AND ("Miniscrew-anchored"):ti,ab,kw OR ("orthodontic miniplates"):ti,ab,kw OR ("orthodontic miniscrews"):ti,ab,kw
**Scopus**	(TITLE-ABS-KEY ("maxillary advancement") OR TITLE-ABS-KEY ("maxillary protraction") OR TITLE-ABS-KEY ("skeletal class III malocclusion") AND TITLE-ABS-KEY ("miniscrew-assisted") OR TITLE-ABS-KEY ("miniscrew-anchored") OR TITLE-ABS-KEY ("orthodontic

 https://doi.org/10.3390/bioengineering12101065

	miniplates") OR TITLE-ABS-KEY ("orthodontic miniscrews"))
**Embase**	('maxillary advancement')/br OR ('maxillary protraction') OR ('skeletal class III malocclusion') AND ('miniscrew-assisted') OR ('miniscrew-anchored') OR ('orthodontic miniplates') OR ('orthodontic miniscrews')

Table S2. Description of PICOs of this systematic review.

**Population (P)**	**Growing Patients in Prepuberal and Puberal Stage with Class III Malocclusion**
Intervention (I)	Maxillary protraction assisted by orthodontic miniplates
Comparison (C)	Maxillary protraction assisted by orthodontic miniscrew
Outcome (O):	Skeletal, dental and soft tissue cephalometric parameters
Study design (s)	Clinical trials, case–control studies, cohort studies, cross-sectional studies, longitudinal studies, prospective studies, and retrospective studies

Table S3. Grading of Recommendation, Assessment, Development, and Evaluation (GRADE) analysis for the studies about treatments with miniplates.

**Study**	**Design**	**Risk of Bias**	**Inconsistency**	**Indirectness**	**Imprecision**	**Publication Bias**	**GRADE Quality**
De Clerck et al. (2010) [9]	Non-RCT	Moderate – based on study design and randomization process	Low – variation across studies	No – applicability to patient population	Low – assessment of sample size and confidence	Unlikely – judged from peer-reviewed status and design	Moderate (●●●○)
Sar et al. (2011) [11]	Non-RCT	Moderate – based on study design and randomization process	Low – variation across studies	No – applicability to patient population	Moderate – assessment of sample size and confidence	Unlikely – judged from peer-reviewed status and design	Moderate (●●●○)
Eid et al. (2016) [22]	Non-RCT	Moderate – based on study design and randomization process	Moderate – variation across studies	No – applicability to patient population	Moderate – assessment of sample size and confidence	Unlikely – judged from peer-reviewed status and design	Moderate (●●●○)
Elnagar et al. (2017) [24]	RCT	Low – based on study design and	Low – variation across studies	No – applicability to patient population	Low – assessment of sample size and	Unlikely – judged from peer-reviewed	High (●●●●)

		randomization process			confidence	status and design	
Bozkaya et al. (2017) [20]	Non-RCT	Moderate – based on study design and randomization process	Low – variation across studies	No – applicability to patient population	Moderate – assessment of sample size and confidence	Unlikely – judged from peer-reviewed status and design	Moderate (●●●○)
Liang et al. (2021) [10]	RCT	Low – based on study design and randomization process	Low – variation across studies	No – applicability to patient population	Low – assessment of sample size and confidence	Unlikely – judged from peer-reviewed status and design	High (●●●●)
Mandall et al. (2024) [26]	RCT	Low – based on study design and randomization process	Low – variation across studies	No – applicability to patient population	Low – assessment of sample size and confidence	Unlikely – judged from peer-reviewed status and design	High (●●●●)

Table S4. Grading of Recommendation, Assessment, Development, and Evaluation (GRADE) analysis for the studies about treatments with miniscrews.

Study	Design	Risk of Bias	Inconsistency	Indirectness	Imprecision	Publication Bias	GRADE Quality
Ge et al. (2012) [21]	RCT	Low – randomization described, but limited info on allocation concealment	Low – similar outcomes between intervention arms	None – comparable groups and outcomes	Some – moderate group sizes, but sufficient effect sizes	Unlikely – well-designed and peer-reviewed	●●●●
Eissa et al. (2018) [23]	Non-RCT	Moderate – non-randomized, possible selection bias	None – consistent skeletal and dental effects	None – appropriate patient population and intervention	Some – small sample size (n=16 per group)	Possible – less rigorous design, but published in peer-reviewed journal	●●●○
Seiryu et al. (2020) [25]	RCT	Low – proper randomization and blinding of outcome assessment	Low – differences between FM and FM+MS group consistent	None – direct comparison of FM vs FM+MS	Low – good power and sample size calculations	Unlikely – randomized trial with registration and ethics approval	●●●●
Kamel et al. (2023) [7]	RCT	Low – proper randomization and	None – consistent results	None – directly applicable to growing	Some – sample size	Unlikely – prospective trial,	●●●●

		intention-to-treat analysis	within the group	Class III patients	limited after drop-outs	peer-reviewed	
